# Avoiding Unnecessary Surgery in Autoimmune Pancreatitis: Lessons From a Four-Patient Case Series and Literature Review

**DOI:** 10.7759/cureus.80356

**Published:** 2025-03-10

**Authors:** Mena Louis, Nathaniel Grabill, Adeel Akhtar, Jay Narula, Angelica Rivera, Edward Foxhall

**Affiliations:** 1 General Surgery, Northeast Georgia Medical Center Gainesville, Gainesville, USA; 2 Surgery, Northeast Georgia Medical Center Gainesville, Gainesville, USA; 3 Internal Medicine, Northeast Georgia Medical Center Gainesville, Gainesville, USA

**Keywords:** autoimmune pancreatitis, biliary stricture, endoscopic ultrasound, ercp, igg4-related disease, obstructive jaundice, pancreatic head lesion, pancreatic mass, steroid therapy

## Abstract

Autoimmune pancreatitis (AIP) is an inflammatory disease that may be mistaken for pancreatic cancer, especially when there is a focal lesion in the pancreatic head. It often involves biliary strictures and occasional tumor marker elevations, causing confusion with malignancy. An incomplete assessment might lead to unwarranted surgery for what is actually an inflammatory process. By combining imaging, histopathology, IgG4 measurement, and the clinical response to steroids, physicians can reach the correct diagnosis more reliably.

We describe four adults, ages 64 to 84, who had obstructive jaundice, biliary dilation, and imaging findings suggesting a possible pancreatic head tumor. Tumor marker levels varied; some were elevated, while others were unremarkable. In two instances, imaging raised concerns about vascular involvement. Endoscopic ultrasound-guided biopsies showed lymphoplasmacytic inflammation without malignant cells, and all patients had elevated IgG4 levels that decreased with steroid therapy.

Each individual underwent endoscopic biliary stenting and began a course of prednisone, leading to a return of normal liver function and improvement in imaging findings. Two required short-term additional stent management for persistent strictures, and three underwent gallbladder removal due to associated disease. All four avoided major pancreatic surgery and had a favorable clinical course.

These cases show the importance of a methodical workup that includes IgG4 assessment and biopsy confirmation. Steroid therapy can resolve clinical and radiologic abnormalities once AIP is recognized. This approach spares many patients from extensive operations when their presumed neoplasm is, in reality, an autoimmune condition.

## Introduction

Autoimmune pancreatitis (AIP) is an inflammatory condition marked by elevated IgG4 levels and distinctive histopathological features [1]. Despite a generally favorable prognosis, it often creates diagnostic dilemmas because it can resemble pancreatic malignancies on imaging [2]. Clinicians may see a focal pancreatic lesion, biliary strictures, or both, making AIP an important consideration when evaluating painless obstructive jaundice [3].

Accurate identification of AIP is crucial. Misinterpreting it as cancer can lead to extensive surgical procedures that are ultimately unwarranted [4]. By combining imaging, IgG4 serology, and histopathological findings, the likelihood of a correct diagnosis increases. In many cases, confirming the disease spares individuals from major resection [5].

Ongoing research has shed more light on AIP’s presentation, pathophysiology, and relationship to other IgG4-related diseases [6]. Many affected patients respond well to corticosteroid therapy, which relieves symptoms, corrects lab abnormalities, and averts long-term complications [7]. Understanding the broad clinical spectrum of this entity remains essential for physicians, surgeons, and radiologists involved in pancreatic disease management.

## Case presentation

Case 1

**Figure 1:**
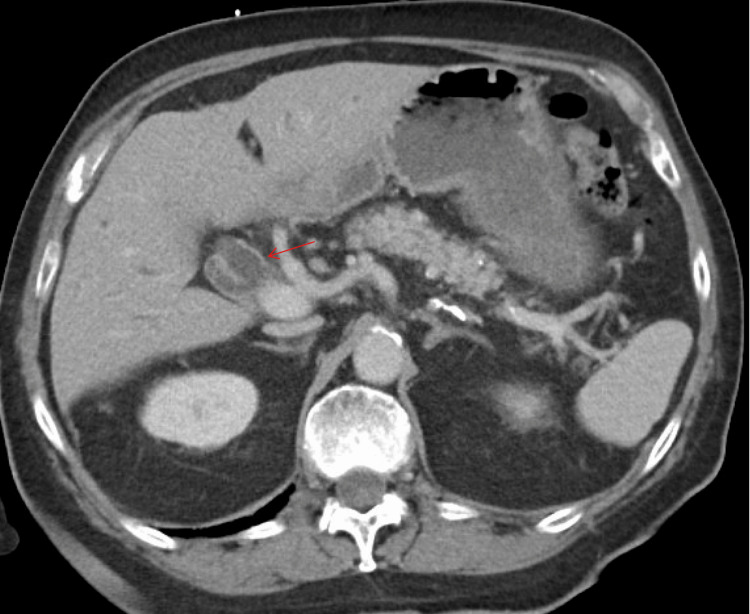
CT abdomen and pelvis, axial view in venous phase. The red arrow points at a moderately dilated common bile duct measuring 15 mm, with associated intrahepatic ductal dilation.

An 84-year-old man presented with dark urine, jaundice, generalized itching, and loose stools. He denied abdominal pain, fever, chills, or significant weight loss. Initial laboratory tests revealed markedly elevated total bilirubin of approximately 18 mg/dL (reference range: 0.1-1.2 mg/dL), alkaline phosphatase above 500 U/L (reference range: 44-147 U/L), and moderately elevated AST and ALT. A CT scan demonstrated dilation of both intrahepatic ducts and the common bile duct without evidence of gallstones (Figure 1). Given his clinical presentation with painless jaundice and cholestatic liver tests, serum IgG4 was measured early and found significantly elevated (>500 mg/dL, reference range: 4-86 mg/dL), raising suspicion for autoimmune pancreatitis (AIP).

An endoscopic evaluation was subsequently performed, initially with ERCP due to logistical constraints and limited availability of the echoendoscope at the time of the procedure. ERCP revealed a high-grade distal common bile duct stricture; balloon sweeps removed thick, dark bile sludge, and a plastic stent was placed to relieve the obstruction. Following an endoscopic retrograde cholangiopancreatography (ERCP), an endoscopic ultrasound (EUS) was performed, identifying an ill-defined hypoechoic mass in the pancreatic head measuring approximately 2.5 cm × 2.0 cm. Fine-needle biopsy demonstrated dense lymphoplasmacytic infiltration with storiform fibrosis, consistent with autoimmune pancreatitis. Immunohistochemical staining confirmed abundant IgG4-positive plasma cells (>50 cells per high-power field, reference <10/HPF). The patient received a brief course of prednisone therapy, resulting in significant improvement of bilirubin and alkaline phosphatase levels without further invasive interventions.

Case 2

**Figure 2:**
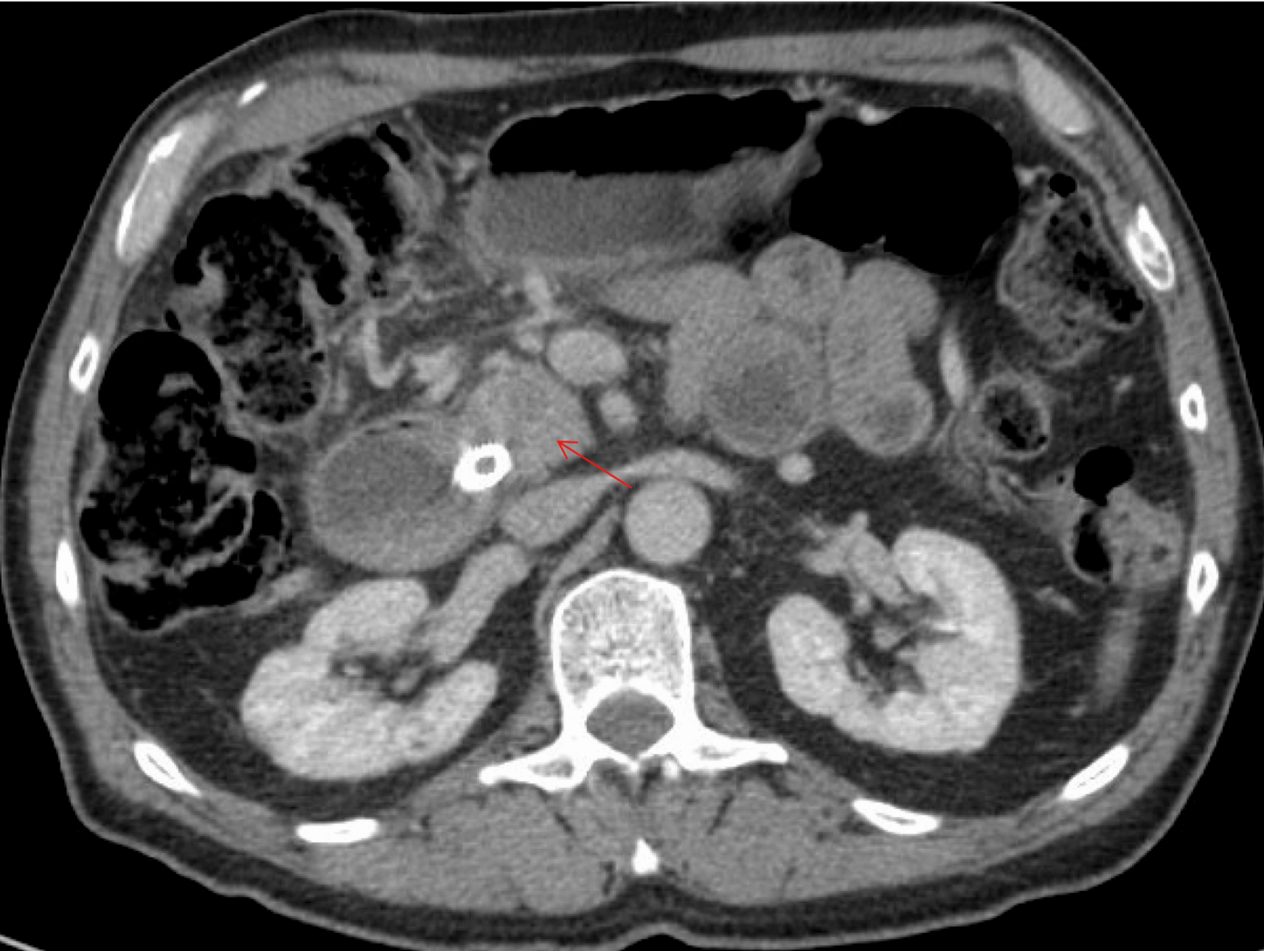
CT chest, abdomen, and pelvis, axial view in venous phase. The red arrow points at a hypoenhancing, rounded mass in the pancreatic head, abutting but not deforming the superior mesenteric vein.

**Figure 3:**
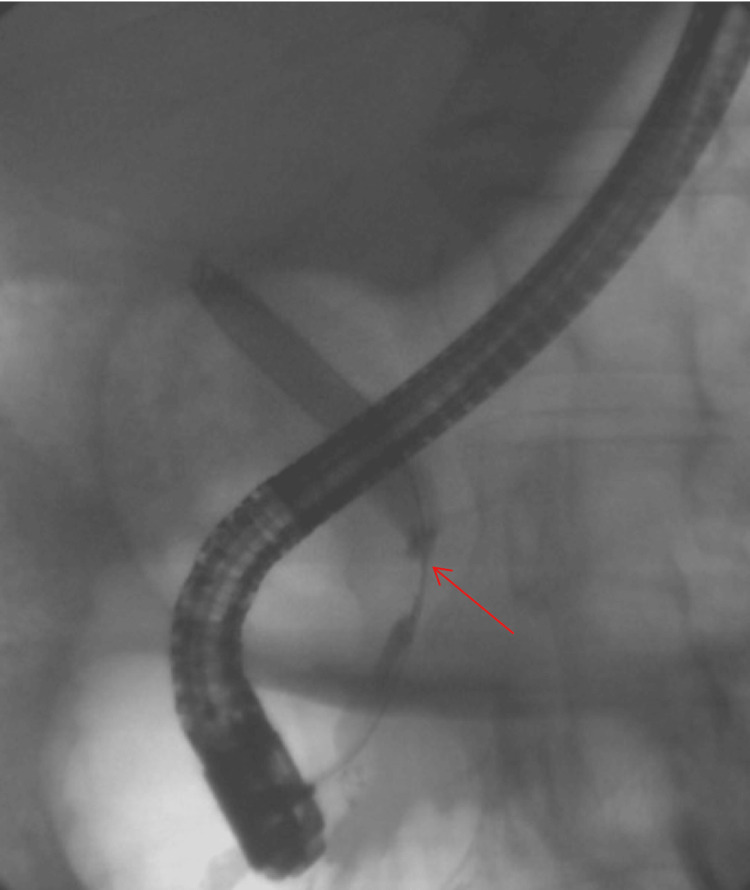
ERCP image. The red arrow points at a narrowing in the distal common bile duct, raising concern for a malignant stricture.

A 66-year-old man presented with progressive, mild epigastric pain radiating to the back and an unintentional 25-pound weight loss. Earlier tests had shown gallbladder issues, prompting a cholecystectomy, yet he continued to have abnormal liver profiles. He underwent a CT scan, which revealed a sizable mass in the pancreatic head with biliary dilation (Figure 2). An ERCP (Figure 3) placed a metallic biliary stent, and a biopsy from the mass initially raised concern for malignancy. Serum IgG4 turned out to be high, and the final pathology showed inflammatory changes consistent with autoimmune pancreatitis. He started steroid therapy, experienced relief from his epigastric discomfort, and regained some of the weight he had lost. Later checks confirmed the resolution of the ductal obstruction, and the stent was removed without incident.

Case 3

**Figure 4:**
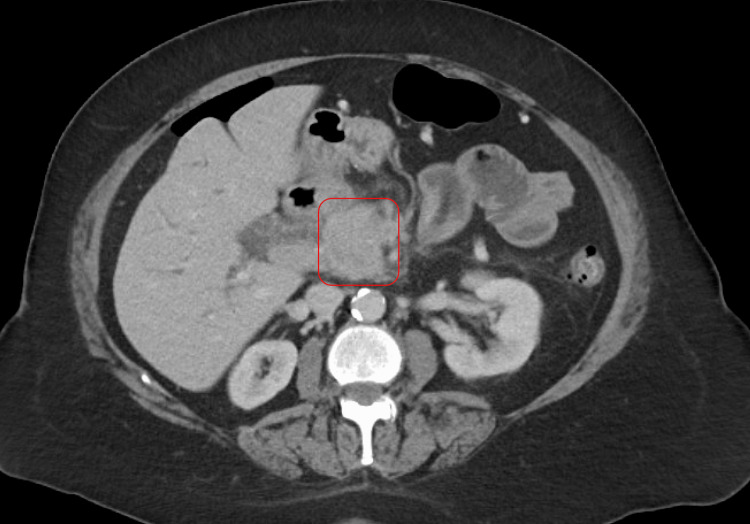
CT abdomen and pelvis, axial view in venous phase. The red arrow points at a pancreatic mass with encasement of the superior mesenteric vein and proximal portal vein.

A 64-year-old woman with a significant smoking history had painless jaundice, upper abdominal discomfort, and a 25-pound weight loss over several weeks. Laboratory studies showed notable elevations in bilirubin, alkaline phosphatase, and transaminases, along with a raised CA 19-9 level. Imaging revealed a 2.8 cm lesion in the head of the pancreas, partial involvement of nearby veins, and a dilated biliary tree (Figure 4). This prompted ERCP with a plastic stent and EUS-guided biopsy. The biopsy showed a dense infiltrate of inflammatory cells and elevated IgG4 staining. She started prednisone, and subsequent imaging demonstrated a reduction in biliary stricture severity. She underwent laparoscopic cholecystectomy because of gallbladder disease, and regular follow-up indicated improved lab results and stable imaging findings.

Case 4

**Figure 5:**
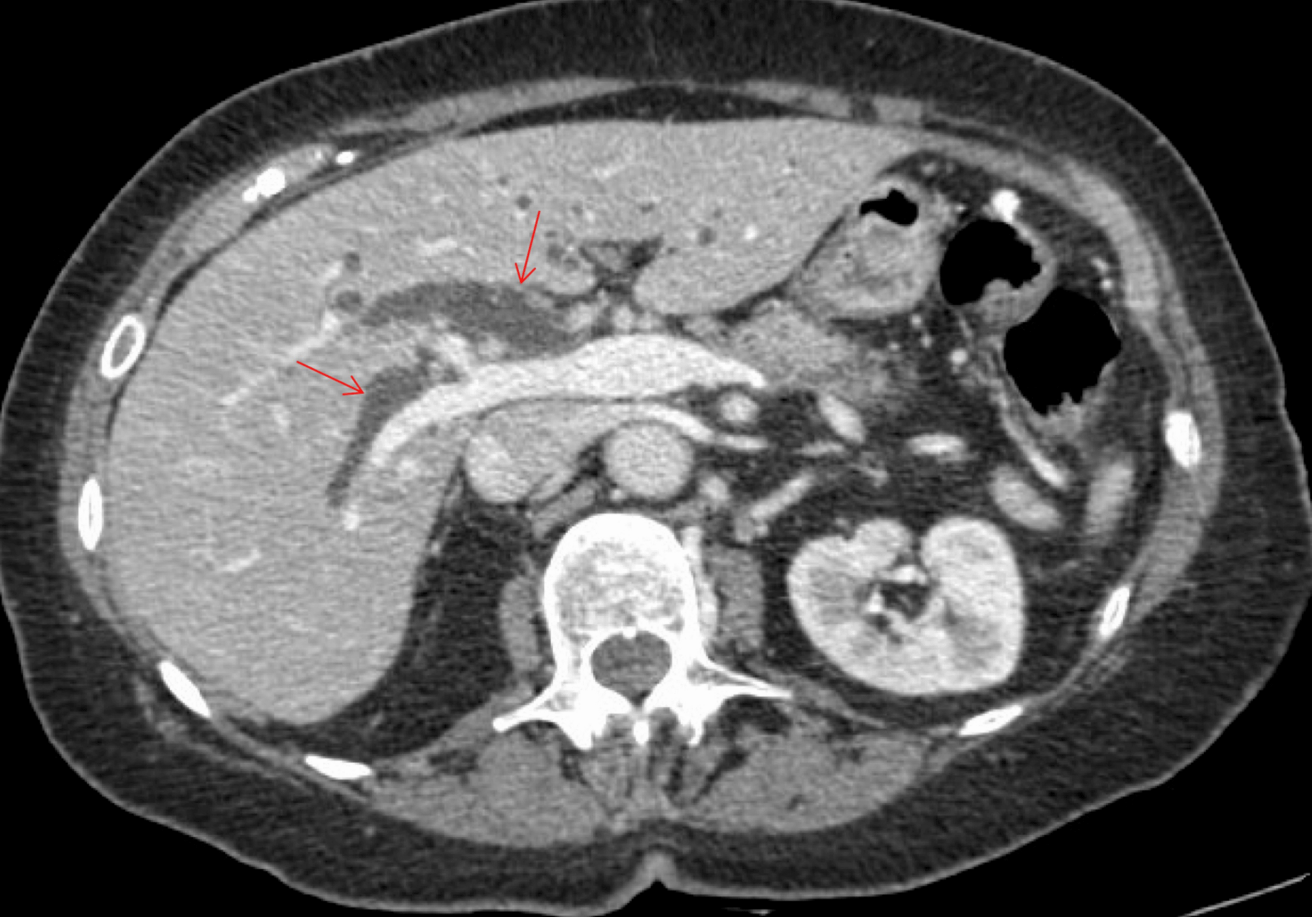
CT abdomen and pelvis, axial view in venous phase. The red arrows point to significant intrahepatic and common bile duct dilation.

An 82-year-old woman with atrial fibrillation, angina, and hearing impairment developed painless jaundice and was found to have a small pancreatic head-uncinate lesion and biliary duct dilation on imaging (Figure 5). Multiple biopsies over two separate procedures showed rare, atypical glands but ultimately no sign of malignancy. She had an elevated IgG4 level and responded to steroid therapy, along with endoscopic placement of a fully covered metal stent for bile duct obstruction. Following successful treatment, her IgG4 normalized, and her stent was removed once imaging confirmed the resolution of the stricture. She was also given enzyme replacement for exocrine insufficiency. Later, she required a laparoscopic cholecystectomy due to gallbladder pathology. Long-term monitoring showed no relapse of jaundice, stable liver function results, and normal IgG4 levels.

## Discussion

Autoimmune pancreatitis (AIP) is recognized as part of the IgG4-related disease spectrum, a fibroinflammatory process capable of affecting multiple organs [1]. AIP can manifest as a focal mass in the pancreatic head, sometimes with biliary obstruction that mimics cholangiocarcinoma or pancreatic adenocarcinoma [2]. The condition presents a major diagnostic challenge because patients can show elevated tumor markers, significant ductal changes, or even vascular encasement on imaging, all of which often point toward malignancy rather than an inflammatory process [3]. Promptly identifying AIP avoids unnecessary major resection.

Several studies have discussed the elevated IgG4 levels associated with Type 1 AIP, often above the conventional cutoff of 135 mg/dL, which was used in earlier diagnostic criteria [4,5]. While this marker is not universally elevated in every patient, the cases presented here all demonstrated IgG4 levels well above normal at some point, reinforcing the role of IgG4 measurement in guiding the diagnostic workup [6]. Histopathological evidence of a lymphoplasmacytic infiltrate and storiform fibrosis further supports the diagnosis, and most patients experience rapid clinical and biochemical improvement once steroid therapy begins [7].

Imaging patterns vary among patients, yet all four examples in this series showed a focal lesion in the pancreatic head. In two instances, cross-sectional studies revealed the involvement of adjacent vessels, and in one case, tumor markers such as CA 19-9 were sufficiently elevated to raise strong concern for cancer. Other published reports note that up to 10% of AIP cases present with CA 19-9 elevations that overlap with ranges commonly seen in malignancy. Thus, clinicians need to interpret tumor markers in the context of biopsy results, IgG4 levels, and clinical response to steroids.

An important issue involves how best to differentiate AIP from pancreatic cancer [8]. ERCP with stent placement can rapidly resolve obstructive symptoms and is indicated regardless of the underlying diagnosis, but obtaining a tissue sample is essential for confirmation [9]. Endoscopic ultrasound-guided biopsy is usually reliable, though repeat sampling may be needed if results are inconclusive [10]. The patients described here benefited from multiple sampling attempts in a few instances before the final determination of AIP could be established.

The favorable responses to steroids in all four patients echo findings in larger studies, where steroid therapy is regarded as a cornerstone of AIP management [11]. Treatment typically results in swift declines in bilirubin, resolution of ductal strictures, and normalization of IgG4. These effects strongly support an inflammatory rather than neoplastic etiology, and early intervention can spare patients the morbidity of extensive surgery [12]. However, some individuals experience relapses or require prolonged immunomodulator regimens, underscoring the importance of long-term follow-up.

Another aspect relates to biliary disease and pancreatic exocrine insufficiency [13]. Three of these patients had gallbladder pathology necessitating cholecystectomy, and two of them required enzyme supplementation. Literature suggests that up to half of AIP cases have associated exocrine dysfunction [14]. These issues often improve after successful steroid treatment but may persist in individuals who have advanced fibrosis [15]. Ensuring adequate nutritional support and controlling symptoms of malabsorption are key components of ongoing care.

This collection of cases underlines the necessity of a structured approach when patients present with a suspected pancreatic head lesion. Imaging alone cannot reliably rule out cancer, and tumor markers have limited specificity. Instead, a combination of clinical presentation, IgG4 measurement, endoscopic sampling, and response to therapy provides a path to accurate diagnosis. These concepts are central to the evolving understanding of AIP and indicate how important it is for clinicians to consider this diagnosis when evaluating an apparent pancreatic tumor with or without elevated tumor markers.

## Conclusions

These four patients collectively show that imaging, IgG4 testing, endoscopic biopsy, and a short course of steroid therapy can differentiate autoimmune pancreatitis from malignancy. By recognizing the autoimmune nature of these masses and managing them with medical therapy, patients avoid extensive surgical procedures. Ongoing imaging and laboratory follow-up remain vital to address possible relapses and maintain stable outcomes.
